# Manipulating CD4+ T Cell Pathways to Prevent Preeclampsia

**DOI:** 10.3389/fbioe.2021.811417

**Published:** 2022-01-12

**Authors:** Eileen J. Murray, Serena B. Gumusoglu, Donna A. Santillan, Mark K. Santillan

**Affiliations:** ^1^ Department of Obstetrics and Gynecology, University of Iowa Carver College of Medicine, Iowa City, IA, United States; ^2^ Department of Psychiatry, Iowa City, IA, United States; ^3^ Institute for Clinical and Translational Science, Iowa City, IA, United States; ^4^ Francois M. Abboud Cardiovascular Research Center, Iowa City, IA, United States; ^5^ Interdisciplinary Program in Molecular Medicine, Iowa City, IA, United States; ^6^ Center for Immunology, University of Iowa, Iowa City, IA, United States

**Keywords:** preeclampsia, CD4+ T cells, prevention, treatment, early pregnancy

## Abstract

Preeclampsia (PreE) is a placental disorder characterized by hypertension (HTN), proteinuria, and oxidative stress. Individuals with PreE and their children are at an increased risk of serious short- and long-term complications, such as cardiovascular disease, end-organ failure, HTN, neurodevelopmental disorders, and more. Currently, delivery is the only cure for PreE, which remains a leading cause of morbidity and mortality among pregnant individuals and neonates. There is evidence that an imbalance favoring a pro-inflammatory CD4+ T cell milieu is associated with the inadequate spiral artery remodeling and subsequent oxidative stress that prime PreE’s clinical symptoms. Immunomodulatory therapies targeting CD4+ T cell mechanisms have been investigated for other immune-mediated inflammatory diseases, and the application of these prevention tactics to PreE is promising, as we review here. These immunomodulatory therapies may, among other things, decrease tumor necrosis factor alpha (TNF-α), cytolytic natural killer cells, reduce pro-inflammatory cytokine production [e.g. interleukin (IL)-17 and IL-6], stimulate regulatory T cells (Tregs), inhibit type 1 and 17 T helper cells, prevent inappropriate dendritic cell maturation, and induce anti-inflammatory cytokine action [e.g. IL-10, Interferon gamma (IFN-γ)]. We review therapies including neutralizing monoclonal antibodies against TNF-α, IL-17, IL-6, and CD28; statins; 17-hydroxyprogesterone caproate, a synthetic hormone; adoptive exogenous Treg therapy; and endothelin-1 pathway inhibitors. Rebalancing the maternal inflammatory milieu may allow for proper spiral artery invasion, placentation, and maternal tolerance of foreign fetal/paternal antigens, thereby combatting early PreE pathogenesis.

## Introduction

Preeclampsia (PreE) is a multisystem hypertensive disorder that affects 5–7% of pregnancies and causes up to 18% of maternal deaths annually in the United States (Harmon et al., 2016; Robertson et al., 2019). This placental disorder is a leading cause of prematurity and low birth weight in babies and costs the US $2.18 billion annually (Harmon et al., 2016; Amaral et al., 2017; Cornelius et al., 2019; Robertson et al., 2019). While PreE often occurs in first pregnancies (4.1% of first pregnancies vs 1.7% in later pregnancies (Hernández-Díaz et al., 2009)), it has a significant rate of recurrence, with a risk of 14.7% following a PreE pregnancy and a risk of 31.9% following two prior PreE pregnancies (Hernández-Díaz et al., 2009). Additionally, PreE is associated with long-term adverse health outcomes, especially in high-risk individuals and their children (Mostello et al., 2008). Despite documentation of the disease and its health implications dating back to almost 400 BCE (Before the Common Era) by Hippocrates, the underlying mechanism(s) and pathophysiology remain poorly understood (Burton et al., 2019). Consequently, prophylactics are limited to lifestyle modification and aspirin, while treatment is limited to delivery (Committee on Practice Bulletins-Obstetrics, 2020). Though early induction of pregnancy is often required when PreE threatens maternal health, there is evidence of significant health consequences following premature (Behrman and Butler, 2007). For example, neonatal respiratory morbidity has been evaluated at 4.4 times greater in pre-term infants than in at-term infants (Khashu et al., 2009).

Preventing PreE and its sequelae involves more than simply lowering maternal blood pressure (Amador et al., 2014; Redman, 1991); novel, molecularly-directed therapeutics are required. Many emerging therapeutic mechanisms of interest are immune in nature, as we review here. Clinical and preclinic studies have demonstrated that successful immunomodulation may prevent and/or treat the root immunologic causes of PreE and other inflammatory diseases. This review will examine therapeutic approaches involving modulation of CD4+ T cell mechanisms and their application to PreE.

## Preeclampsia Diagnosis and Management

According to American College of Obstetrics and Gynecology (ACOG) guidelines, which aligns with the International Society for the Study of Hypertension (ISSHP) guidelines, the clinical diagnosis of PreE, which is made only after the 20th week of gestation, requires pregnancy-specific hypertension (>140/90 2 separate occasions at least 4 h apart) and one of the following: proteinuria; low platelets; diseased liver, kidney, or lung; or new-onset headaches or visual problems. PreE with severe features is further diagnosed if systolic and diastolic blood pressure exceed 160 and 110 mmHg, respectively; central nervous system dysfunction (e.g., photophobia, severe headache) occurs; or in instances of thrombocytopenia or hepatic, renal, or pulmonary signs (Brown et al., 2018; Committee on Practice Bulletins-Obstetrics, 2020). When PreE complicates preexisting HTN (seen in 20–50% of pregnancies with chronic HTN), it is considered superimposed (Brown et al., 2018; Committee on Practice Bulletins-Obstetrics, 2020). PreE has a large spectrum of clinical presentations, and may present in individuals with or without existing hypertension, metabolic disease, obesity, genetic variants, or environmental risk factors (Wallis et al., 2008). Additionally, PreE is a highly clinically heterogeneous diagnosis, with clinical presentations that can include more canonically pro-inflammatory phenotypes, as well as more metabolic or endocrine phenotypes, as have been defined in molecular studies (Leavey et al., 2015). The clinical diagnosis and management of PreE is therefore highly complex, requiring significant expertise, experience, and sound judgement.

PreE, both with and without severe features, results in chronic immune activation, endothelial dysfunction, and often intrauterine growth restriction and fetal growth restriction (FGR). PreE risk factors include chronic HTN, chronic inflammatory medical conditions, obesity, family or personal history, genetic predispositions (e.g. mutations in RGS2) (Perschbacher et al., 2020), primiparity or new paternity (Li and Wi, 2000; Saftlas et al., 2003; Duckitt and Harrington, 2005), and low paternal exposure due to barrier contraceptives or a short interval between first coitus and conception (Duckitt and Harrington, 2005; Kho et al., 2009). Though PreE may progress even beyond delivery of the placenta, its initiation is often associated with early placentation processes.

## Preeclampsia Results in Serious Short- and Long-Term Health Complications

Both the immediate and long-term health impacts of PreE are significant. PreE is responsible for over 70,000 maternal and 500,000 fetal/neonatal deaths globally (Duley, 2009). Serious immediate maternal morbidities are associated with PreE and include stroke; HTN; shock; seizure (eclampsia); acute respiratory syndrome; coagulopathy; elevated liver enzymes and low platelets (HELLP) syndrome; renal, pancreatic, or liver failure; and pulmonary edema (Duley, 2009; Amaral et al., 2017; Mayrink et al., 2018; Committee on Practice Bulletins-Obstetrics, 2020).

Individuals with a history of PreE are at particularly increased risk of serious cardiovascular complications after placental delivery, including HTN, myocardial infarction, stroke, venous thromboembolism, pulmonary edema, acute respiratory syndrome, intraventricular hemorrhage, sepsis, and bronchopulmonary dysplasia, and more (Mayrink et al., 2018; Committee on Practice Bulletins-Obstetrics, 2020). In fact, the 2011 American Heart Association guidelines for prevention of cardiovascular disease (CVD) in women equates PreE to chronic HTN, diabetes mellitus (DM), and obesity in terms of risk for future CVD (Roger et al., 2011). One in five patients develop HTN within 7 years of PreE, compared to only 2% after uncomplicated pregnancies (Bellamy et al., 2007).

Fetal mortality is also significantly increased with PreE (Santillan et al., 2009), and babies born to PreE pregnancies have increased risk for FGR and preterm birth risk (Duley, 2009). Additionally, children of PreE pregnancies have an increased risk of neurodevelopmental or behavioral conditions and CVD as they grow up, on top of the immediate risks of prematurity (Amaral et al., 2017; Gumusoglu et al., 2020).

## Preeclampsia Pharmacotherapy

Pharmacologic treatments for PreE or its prevention are lacking, and management of PreE is focused on symptom alleviation and maintenance of the pregnancy until 37 weeks or sooner if there are more severe signs of PreE. After 37 weeks, removal of the placenta resolves PreE in most cases: only 18.4% of postpartum PreE occurs in patients with an antecedent diagnosis of PreE (Al-Safi et al., 2011).

Prevention of PreE and associated morbidities is currently limited to aspirin. A recent meta-analysis of 45 randomized trials revealed that, when initiated before 16 weeks, a low dose of aspirin (LDA) significantly reduced PreE (risk ratio, RR, 0.47; confidence interval, CI 0.43–0.75), severe PreE (RR, 0.47; CI, 0.26–0.83), and FGR (RR, 0.56; CI, 0.44–0.70) (Roberge et al., 2017; Roberge et al., 2018). However, a LDA initiated after 16 weeks of gestation was not associated with reduced risk of severe PreE (RR, 0.85; CI, 0.64–1.14) or FGR (RR, 0.95; CI, 0.86–1.05), and only with a small reduction in PreE risk (RR, 0.81; CI, 0.66–0.99) (Roberge et al., 2017; Roberge et al., 2018). Therefore, LDA therapy is unlikely to significantly curtail global PreE-associated morbidity and mortality rates.

Another common PreE pharmacotherapy, magnesium sulfate, only prevents eclamptic seizures, which are rare. Similarly, PreE treatment with antihypertensives does not reverse disease processes (Duley et al., 2010). Some antihypertensives are even contraindicated in pregnancy, per the US Food and Drug Administration (FDA) category X labeling, including angiotensin-converting enzyme (ACE) inhibitors and angiotensin II receptor blockers (ARB) due to potential teratogenic and fetotoxic effects (Redman, 2011). Given these limitations, novel classes of PreE pharmacotherapy are required. An emerging area of study is the immunologic prevention of PreE, which is based largely on CD4+ T cell mechanisms.

## CD4+ T cells in Pregnancy and Preeclampsia

As has been expertly reviewed elsewhere, CD4+ T cells and their immune products play a critical role in regulating normal reproductive processes. For instance, proper T cell responses govern early placentation, fertilization, and embryogenic processes, including angiogenic and patterning cascades (Trowsdale and Betz, 2006; Ingman and Jones, 2008; Howerton and Bale, 2012; Krishnan et al., 2013; Göhner et al., 2017). At the maternal-fetal interface, CD4+ T cells regulate dendritic cell maturation, NK cell cytolytic behavior, and the behavior of a host of cell types (e.g., placental trophoblasts, vascular endothelium, etc.) via release of pro- and anti-inflammatory cytokines and chemokines. However, these normative immune cell processes may go awry, increasing risk for PreE, as we detail in the present review. Therapeutic approaches for modulating CD4+ T cell polarization towards increased type 2 helper CD4+ T cells (Th2) and regulatory T cells (Tregs) are thus promising for PreE given pro-inflammation during early placental morphogenesis is a pathogenic risk factor for PreE. Altered immunology is a pervasive problem in PreE, beginning as early as conception (Robertson et al., 2019). Advanced modeling and analytic methods offer some promise for the detection of high-risk pregnancies (Fischer and Voss, 2014). Targeting high-risk pregnancies and then ameliorating this immunologic imbalance early in disease progression may prevent subsequent oxidative stress and poor placentation processes. If applied to high-risk pregnancies, early prevention may reverse or slow PreE pathogeneses before symptoms arise.

CD4+ T cells are a common denominator in the immunologic mechanisms underlying PreE, linking multiple pathways that may be targeted by intervention. Furthermore, therapies affecting dendritic cells (DCs), natural killer (NK) cells, B cells, neutrophils, and other immune cells ultimately impact upstream the CD4+ T cell counterparts. Preclinical data in animal models of PreE, as well as human studies in other immune-mediated diseases characterized by chronic inflammation, reveal multiple effective therapeutic approaches to manipulating CD4+ T cell mechanisms to restore a healthy balance. Further developing these therapies for use in PreE will provide options to minimize PreE’s global health burden.

## Two Stage Model of Preeclampsia

Redman in 1991 described the Two Stage Model which is a traditional explanation of the development of PreE (Redman, 1991). This model consists of two stages: pre-clinical and clinical (Redman, 1991; Redman et al., 1999; Staff, 2019). Stage 1 is defined by poor placentation and altered syncytiotrophoblast function in the outermost placental layer, which is made of epithelial cells that invade the wall of the uterus during placentation. During this stage, there is an excessive pro-inflammatory immune response due to failed maternal immune tolerance, which leads to inappropriate uterine spiral artery remodeling, placental hypoperfusion, hypoxia, and oxidative stress. Stage 2 is characterized by clinical symptoms (e.g., HTN and proteinuria), which arise from biological stressors that occur during stage 1 (e.g.,syncytiotrophoblast microvillous fragmentation, hypoxic damage, oxidative stress) (Redman, 2013).

**FIGURE 1:**
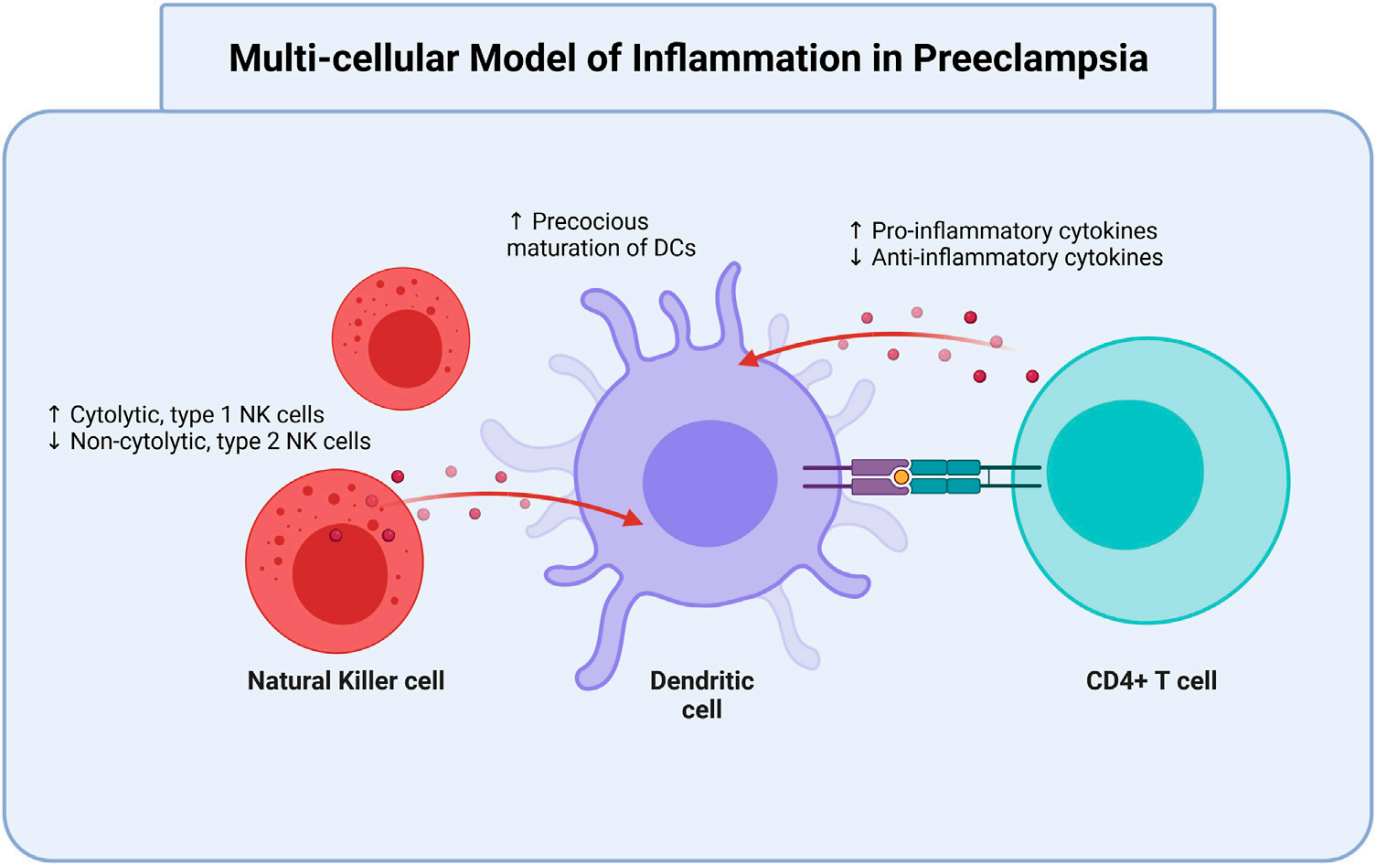
Dendritic cells are modulated by T cells and NK cells in preeclampsia. Oxidative and vascular stress contribute to preeclamptic conditions. In preeclampsia, there is an increase of cytolytic, type 1 NK cells and precocious maturation of DCs, as well as increased production of pro-inflammatory cytokines. There is a simultaneous decrease in non-cytolytic, type 2 NK cells and anti-inflammatory cytokines. All three cell types interact to aggravate the elevated pro-inflammatory status that contributes to and occurs in preeclampsia. NK, natural killer; DC, dendritic cells. Created with https://biorender.com.

The pre-clinical stage 1 of PreE occurs through approximately week 20 of gestation (Redman and Sargent, 2010), during which shallow cytotrophoblast (cells which differentiate into syncytiotrophoblasts and other placental cells) migrate towards the uterine spiral arterioles. This invasion contributes to inadequate spiral artery remodeling in early pregnancy. Without proper invasion and arterial remodeling, there is pulsatile high-pressure blood flow in the placental arteries and therefore placental oxidative stress and poor supplementation of the intervillous space (Burton et al., 2009). Placental ischemia is associated with reperfusion, apoptosis, macromolecule release [e.g. placental (PLGF) and vascular endothelial (VEGF) growth factors] into maternal circulation, and finally promotion of intravascular inflammatory response factors and free radicals associated with maternal vascular dysfunction (Mayrink et al., 2018). These changes include increased endothelin-1 (ET-1), antiangiogenic factor soluble Fms-like Tyrosine Kinase-1 (sFlt-1), agonistic autoantibodies to angiotensin II type 1 receptor (AT1-AA), and decreased nitric oxide (NO). The combination of these vasoconstrictive factors further prompts increased levels of pro-inflammatory immune cells and cytokines (Borzychowski et al., 2006; Harmon et al., 2016; Amaral et al., 2017; Staff, 2019; Aneman et al., 2020). Furthermore, increased pro-inflammatory type 1 helper CD4+ T cells (Th1) and type 17 helper CD4+ T cells (Th17) is met with decreased anti-inflammatory Th2 and Tregs. a multi-cellular model of inflammation in PreE best illustrates the imbalanced cellular interactions that contribute to PreE pathogenesis (Figure 1).

The second stage of PreE is the clinical manifestation of systemic maternal disease. Stage 2 occurs during the second half of pregnancy and is defined by placental hypoxia and oxidative stress, clinical HTN, and proteinuria secondary to vascular inflammatory dysfunction and spiral artery insufficiency (Redman, 1991; Burton et al., 2019; Staff, 2019). While a discrete factor linking stage 1 to stage 2 is unknown, maternal immunological response to the fetus is one possible explanation. Targeting immune mechanisms in PreE therefore provides an opportunity to prevent clinical disease progression from the outset.

## Immunology of Preeclampsia

Though PreE development is impacted by multiple factors and pathways, immunologic mechanisms offer promise for prevention because of the early and pervasive impacts of inflammation in PreE. An abnormal immune response and high Th1/17:Th2/Treg ratio is “central and causal” to PreE, as has been expertly reviewed elsewhere (Redman et al., 1999; Sargent et al., 2006; Saito et al., 2007; Sasaki et al., 2007; Robertson et al., 2019). The immune response leading to PreE may start as early as conception, as contact with paternally-derived transplantation antigens during coitus and conception primes Treg activation (Robertson et al., 2019). Proposedly, discrimination of paternal/fetal cells as non-self by the uterine immune system mounts an inflammatory offense that contributes to the broader pro-inflammatory immune imbalance in PreE (Robertson et al., 2019).

In healthy pregnancies, a pro-inflammatory CD4+ T cell response promotes trophoblast invasion, subsequent placentation, and an anti-inflammatory response, thus inducing local maternal tolerance for the semi-allogeneic fetus and placenta (Scroggins et al., 2018; Burton et al., 2019; Robertson et al., 2019; Staff, 2019; Aneman et al., 2020). During initial placentation, Tregs are at their most prevalent (Harmon et al., 2016). Tregs are partially responsible for shifting the decidual milieu from pro-to anti-inflammatory, thereby promoting immune tolerance in gestation (Robertson et al., 2019).

A carefully regulated, dynamic balance between pro- and anti-inflammatory factors is critical to multiple pregnancy processes. For instance, while an anti-inflammatory response is necessary to allow trophoblast cells to reach and invade the endometrium (Harmon et al., 2016), regulated pro-inflammation is essential to recruit NK cells and macrophages to the decidua to facilitate deep cytotrophoblast invasion into myometrial segments during maternal vascular remodeling (Redman and Sargent, 2005). Adequate spiral uterine remodeling in healthy pregnancy promotes low resistance blood flow via the placental intervillous space, which supplies the fetus with oxygen and nutrients (Aneman et al., 2020), when the pregnancy requires an enhanced circulatory capacity during the second trimester (Redman and Sargent, 2005). Overall, balanced immunity is important for successful placentation, vascular remodeling, and maternal-fetal tolerance.

In PreE, a mild systemic inflammatory response that presents in healthy pregnancies is over-intensified (Borzychowski et al., 2006; Saito et al., 2007; Staff, 2019). There is a shift away from anti-inflammatory cells and cytokines [e.g., Treg, Th2, interleukin (IL)-10, and IL-4] towards pro-inflammatory ones (e.g., Th1, Th17, and IL-4) (Borzychowski et al., 2006; Cornelius, 2018). An elevated Th1:Th2 ratio involves increased secretion of pro-inflammatory cytokines, such as tumor necrosis factor alpha (TNF-α), IL-6, and IL-17 (Harmon et al., 2016; Scroggins et al., 2018). An overactivated systemic inflammatory response then leads to an increase in antiangiogenic factors and placental insufficiency. Underlying mechanisms for this include excess placental oxidative stress and vascular damage, production of angiotensin II AT -1 receptor and sFlt-1 auto-antibodies, and decreased VEGF and PLGF (Gathiram and Moodley, 2016).

Decreased Tregs and increased Th17 CD4+ T cell subtypes may be specifically involved in the pathophysiology of PreE (Cornelius et al., 2015). Many pregnant individuals with PreE have fewer and less functionally competent Treg cells, and a decrease in Treg cells may be proportional to PreE severity (Robertson et al., 2019). The multi-cellular model of inflammation in PreE, demonstrating interactions between DCs, CD4+ T cells, and NK cells (Figure 1). Ultimately, inappropriate activation and subsequent pro- and anti-inflammatory response dysregulation contributes to improper maternal vascular remodeling and shallow trophoblast invasion, as well as endothelial and placental dysfunction in PreE (Harmon et al., 2016; Cornelius, 2018; Aneman et al., 2020).

The mechanisms driving inflammation in PreE are incompletely understood. One possibility is maladaptation to fetal/paternal alloantigens and a lingering immune attack against foreign fetal/paternal antigens (Redman and Sargent, 2010). Changing paternity may increase the risk of PreE by 30%, which supports the allogenic response hypothesis of PreE (Li and Wi, 2000; Redman and Sargent, 2010). Work by Saftlas et al., 2003 demonstrated a protective effect of previous fetal loss due to PreE against subsequent PreE, but only when the same partners are involved in a subsequent pregnancy. Furthermore, the more pre-conceptual exposure to semen, the lower the risk of PreE (Redman and Sargent, 2010). When the same partner is involved, the risk of PreE appears to be higher with a shorter interval between first coitus and conception (Kho et al., 2009). Together, these studies suggest that the chronicity of maternal immune exposure to paternal antigens modulates preeclampsia risk, further demonstrating the potential utility of immunomodulatory preeclampsia therapeutic.

Specifically, therapeutics that guide CD4+ T cell polarization may serve as promising tools in preventing oxidative stress and poor placentation upstream of clinical PreE manifestation. As we review here, preclinical data in similar immune mediated diseases—and in models of PreE itself—demonstrate the potential efficacy of CD4+ T cell modulators in PreE prevention.

## CD4+ T cell pathways are therapeutic targets in multiple inflammatory diseases

**FIGURE 2:**
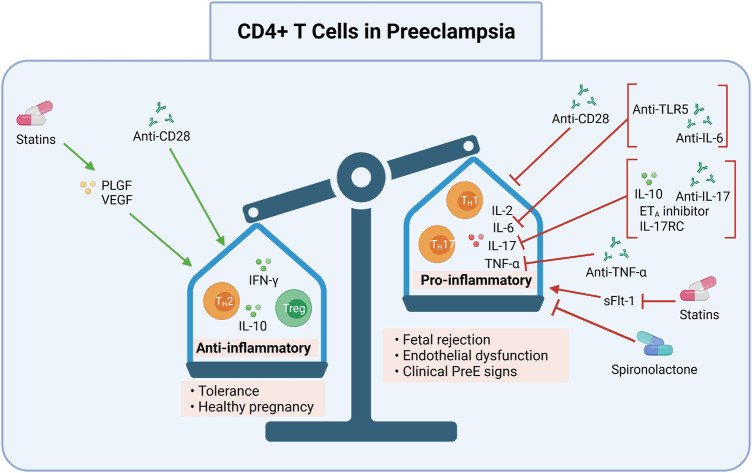
CD4+ T cell pro-inflammation and processes may be targeted in preeclampsia. Induction of anti-inflammatory CD4+ T cells (Th2s) and Tregs, and inhibition of pro-inflammatory CD4+ T cells (Th17s, TH1s) may be one pathway for preeclampsia treatment and prevention. Collectively, modulation of these processes may ameliorate the overly pro-inflammatory immune response in preeclampsia. Shifting the ratio of pro-to anti-inflammatory CD4+ T cells may prevent preeclampsia by allowing proper placentation and reducing resultant oxidative stress. This can be done by 1) promoting an anti-inflammatory milieu (Th2, Treg, IFN-γ, IL-10) with anti-CD28, statins that induce PLGF and VEGF, or 2) inhibiting a pro-inflammatory milieu (Th1, Th17, IL-2, IL-6, IL-17, and TNF-α) with anti-CD28 or spironolactone, or inhibiting sFlt-1using statins. Specific cytokines may also be inhibited, such as IL-6 by anti-TLR5 or anti-IL-6; IL-17 by IL-10, ET_A_ inhibitors, IL-17RC, and anti-IL-17; or TNF-α by anti-TNF-α. The anti-inflammatory CD4+ T cell response may result in tolerance and a healthy pregnancy while the pro-inflammatory CD4+ T cell response may lead to fetal rejection, endothelial dysfunction, and clinical preeclampsia signs. DC, dendritic cells; mAbs, monoclonal antibodies; Th, Helper T cell; Treg, regulatory T cell; IL, interleukin; IFN-γ, interferon gamma; TNF-α, tumor necrosis factor alpha; anti, antibody; ET_A_, endothelin-1 receptor A; sFlt-1, soluble Fms-like Tyrosine Kinase-1; PLGF, placental growth factor; VEGF, vascular endothelial growth factor; IL-17RC mouse IL-17 receptor C; TLR5, toll like receptor 5. Created with https://biorender.com.

**FIGURE 3:**
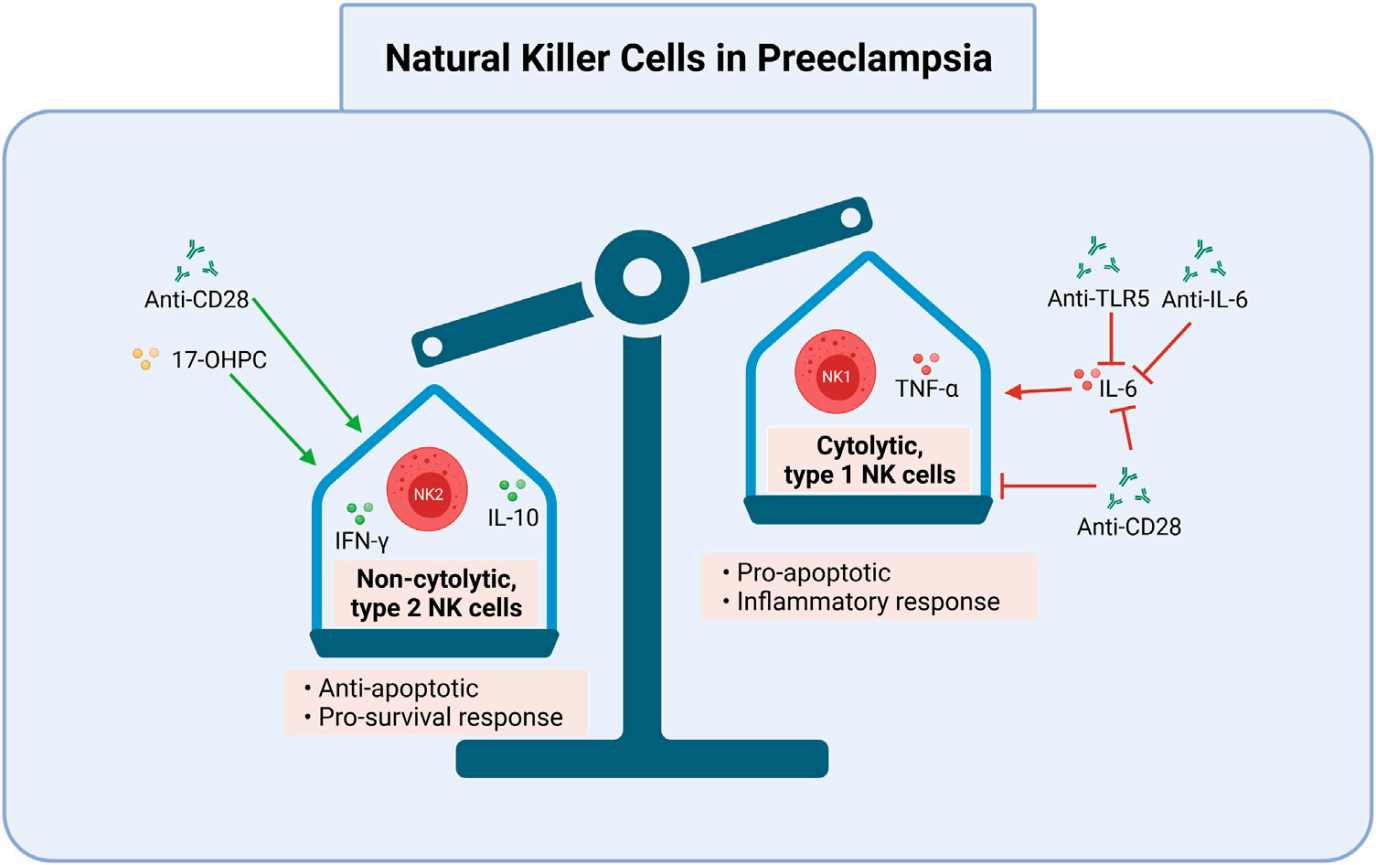
Cytolytic NK cells may be targeted in preeclampsia. Induction of non-cytolytic NK cells and inhibition of cytolytic NK cells is one potential avenue for the treatment and prevention of preeclampsia. Anti-TLR5, anti-IL-6, and anti-CD28 inhibit IL-6’s activation of pro-apoptotic cytolytic type 1 NK cells. Anti-CD28 also directly inhibits type 1 NK cells, and promotes non-cytolytic, type 2 NK cells and the anti-apoptotic cytokines IL-10 and IFN-γ. 17-OHPC works similarly to promote type 2 NK cells. Together, these reduce the cytolytic NK cell response, which can lead to improper placentation and subsequent preeclampsia. 17-OHPC, 17-hydroxyprogesterone caproate; anti, antibody; IFN-γ, interferon gamma; NK2, non-cytolytic, type 2 Natural Killer cells; IL, interleukin; NK1, cytolytic, type 1 Natural Killer cells; TNF-α, tumor necrosis factor alpha; TLR5, toll like receptor 5. Created with https://biorender.com.

**FIGURE 4:**
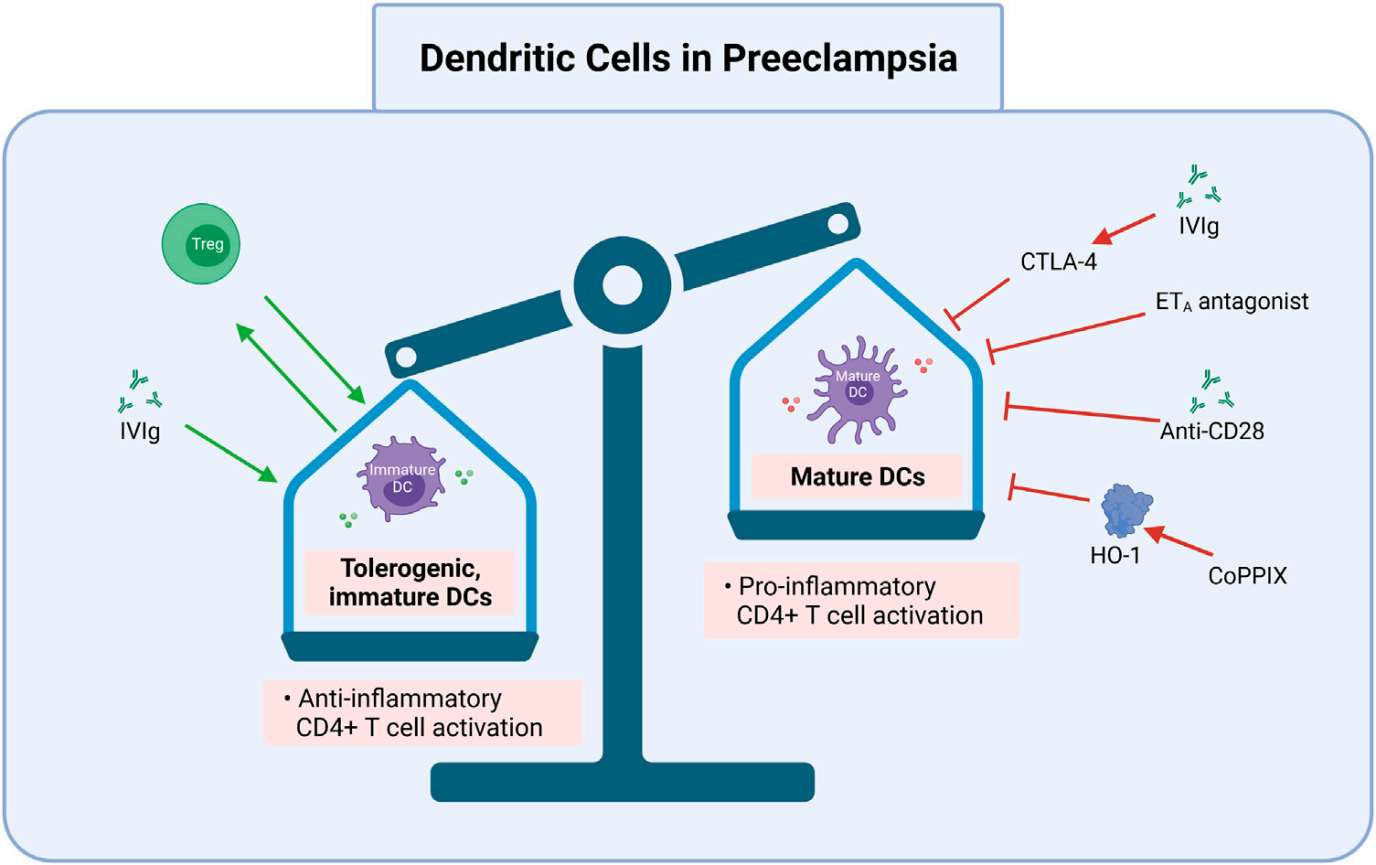
DC cells maturation and function may be targeted in preeclampsia. Inhibition of mature CDs and induction of tolerogenic, immature DCs is one promising route for preeclampsia treatment and prevention. Targets to achieve this include exogenous Treg transfer, IVIgs to promote tolerogenic DCs (e.g., via CTLA-4), CoPPIX induced HO-1 activation, and ET_A_ antagonists. IVIg via CTLA-4, anti-CD28, ET_A_ antagonists, and CoPPIX via HO-1 inhibit mature DC function, which can be pro-inflammatory to CD4+ T cells. Treg, regulatory T cell; DC, dendritic cell; CTLA-4, cytotoxic T lymphocyte-associated antigen-4; IVIg, intravenous immunoglobulin G; ET_A_, endothelin-1 receptor A; anti, antibody; CoPPIX, cobalt protoporphyrin; HO-1, heme oxygenase 1. Created with https://biorender.com.

Preclinical studies demonstrate disease reduction following adoptive exogenous Treg therapy, intrinsic Treg expansion, and decreased pro-inflammatory cytokines via various approaches. Several clinical studies and approved therapies involve CD4+ T cell pathway manipulations across multiple immune-mediated, inflammatory diseases. These diseases include rheumatoid arthritis (RA), Crohn’s disease (CD) and inflammatory bowel disease (IBD), atherosclerosis (AS), DM, pulmonary arterial hypertension (PAH), and systemic lupus erythematosus (SLE). The following therapy target candidates and their respective effects are summarized in Table 1 and in Figures 2, 3, 4.

**TABLE 1:** Immunomodulation offers promise for preeclampsia (PreE) prevention. This table describes potential preeclampsia prevention targets, the directionality of target abnormality in PreE, drugs that modulate the targets, and the effects of each drug on PreE-related biological processes. Treg, regulatory T cell; NK, natural killer; DC, dendritic cell; CTLA-4, cytotoxic T lymphocyte-associated antigen-4; IVIg, intravenous immunoglobulin G; ET_A_, endothelin-1 receptor A; anti, antibody; CoPPIX, cobalt protoporphyrin; HO-1, heme oxygenase 1, mAbs, monoclonal antibodies; Th, Helper T cell; Treg, regulatory T cell; IL, interleukin; IFN-γ, interferon gamma; TNF-α, tumor necrosis factor alphasFlt-1, soluble Fms-like Tyrosine Kinase-1; PLGF, placental growth factor; VEGF, vascular endothelial growth factor; IL-17RC mouse IL-17 receptor C; 17-OHPC, 17-hydroxyprogesterone caproate; NK2, non-cytolytic, type 2 Natural Killer cells; NK1, cytolytic, type 1 Natural Killer cells; TLR5, toll like receptor five; AT1-AA, Angiotensin II Type 1 Receptor Agonistic Autoantibody.

Target	Change in targets in preeclampsia	Drug	Drug effect on preeclampsia mechanisms	Citation
TNF-α	Increase	anti-TNF-α neutralizing mAb	Less pro-inflammatory T cell differentiation via DC maturation and IL-10-dependant inhibition of CD4+ T cell expansion	Yuan et al. (2015), Alijotas-Reig et al. (2017)
			Decreased vasoconstriction, coagulation, vascular permeability, and microvascular leakage	LaMarca et al. (2005), Kaplanski et al. (1997)
			Decreased endothelial dysfunction; trophoblastic apoptosis inactivation	Alexander et al. (2001), Huppertz and Kingdom (2004), Chen et al. (2010), Ibrahim et al. (2017)
CD28	Decrease	CD28 superagonist TGN1412/TAB08	Treg promotion	Tabares et al. (2014)
			Reduced pro-inflammatory response	Ibrahim et al. (2017)
			Reduced cytolytic NK cell activation during placental invasion	Ibrahim et al. (2017)
IL-10	Decrease	IL-10 administration	Promote macrophage maturation to anti-inflammatory M2	Harmon et al. (2015)
Treg	Decrease	adoptive transfer	Promote mast cell repair of the placental and vascular defects	Maganto-García et al. (2011), Matrougui et al. (2011), Woidacki et al. (2015)
			Reduced oxidative stress	Jafri and Ormiston (2017), Dall'Era et al. (2019), Chen et al. (2010)
IL-6	Increase	anti-TLR-5	Increased anti-inflammation	Narazaki et al. (2017)
		anti-IL-6		Tousoulis et al. (2016), Narazaki et al. (2017), Trivedi and Adams (2018)
IL-17	Increase	anti-IL-17	Prevents offspring neurodevelopmental defects	Amador et al. (2014), Reed et al. (2020)
			Improved vascularization; decreased AT1-AA and oxidative stress	Amador et al. (2014)
		ET_A_ antagonists	Downregulated mature DCs; decreased vascular permeability and vasoconstriction	Alexander et al. (2001); Tanaka et al. (2014); Morris et al. (2016)
		IL-17RC	Improved vascularization; decreased IL-17 activation of cytolytic NK cells and decreased pro-inflammation	Cornelius et al. (2013); Travis et al. (2020)
		Spironolactone	Decreased Th17 activation and increased FoxP3-dependent Treg differentiation	Amador et al. (2014)
			Improved placentation via competitive binding of aldosterone	Birukov et al. (2019)
HMG-CoA reductase		Statins	Improved placentation; reduced sFlt-1; increased pro-survival/antiapoptotic factors; upregulated PLGF and VEGF	Youssef et al. (2002); Kumasawa et al. (2011); McDonnold et al. (2014); Esteve-Valverde et al. (2018); 82
Cytolytic NK cells	Increase	17-OHPC	Decreased pro-inflammation	Gupta and Roman (2012); Elfarra et al. (2020)
		CD28 mAbs	Decrease IL-6/IL-2-mediated activation of cytolytic NK cells	Ibrahim et al. (2017)
DCs	skewed towards pro-inflammatory	CTLA-4	Decreased DC maturation and subsequent pro-inflammatory T cell and cytolytic NK cell activations	Gu et al. (2012)
		CoPPIX-mediated HO-1 induction		
		IVIg		Bayry et al. (2003)

### Neutralizing TNF-α

One possible target for CD4+ T cell modulation is TNF-α, which is secreted by Th1 and Th17 cells. TNF-α levels can be two to three times higher in PreE than in normotensive pregnancies (Harmon et al., 2016; Rambaldi et al., 2019) and have been linked to gestational HTN, endothelial dysfunction, and poor obstetric outcomes (Small et al., 2016). Though crucial for correct implantation and placentation (Yuan et al., 2015), TNF-α is attributed to IL-10-dependant inhibition of CD4+ T cell expansion (Alijotas-Reig et al., 2017), preventing a healthy pro- and anti-inflammatory CD4+ T cell balance. Additionally, TNF-α indirectly causes vasoconstriction after initiating systemic pro-inflammatory and pro-apoptotic signaling cascades that increase reactive oxygen species (ROS) and contribute to placental oxidative stress (Redman et al., 1999). Inhibiting TNF-α using anti-TNF-α neutralizing monoclonal antibodies (mAbs) promotes the proliferation of Tregs while suppressing effector T (Teff) cells (Xiao et al., 2021).

Guidelines from the FDA indicate that anti-TNF-α treatments are category B drugs (i.e. drugs that have failed to demonstrated risk to the fetus but for which no well-controlled studies exist in pregnant women) (Raja et al., 2012). The British Association of Dermatologists recommends the use of TNF-α inhibitors on a case-by-case basis in pregnancy (Johansen et al., 2018), and the European Crohn’s and Colitis Organization has found that TNF-α inhibitors may be used safely until the third trimester (Johansen et al., 2018). Because of its presumed safety, anti-TNF-α may be a viable PreE therapy to increase Treg cell populations and rebalance the Th1/Th17:Treg/Th2 ratio in PreE.

Therapeutics targeting TNF-α have also been used in other inflammatory diseases to combat immune dysregulation. For instance, anti-TNF-α neutralizing mAbs are approved for use in RA, AS, and IBD. These drugs include etanercept (Aronson, 2006), infliximad (Wall et al., 1999), certolizumab pegol (Weber-Schöndorfer et al., 2015; J. Egan and Maaser, 2009), golimumab (Taylor et al., 2015), and adalimumab (Taylor et al., 2015; Jafri and Ormiston, 2017; Xiao et al., 2021).

Work in PreE animal models further suggests the utility of TNF-α therapeutics in clinical PreE. For instance, the TNF-α ligand trap etanercept prevents HTN and decreases ROS-induced vascular dysfunction in the reduced uterine perfusion pressure (RUPP) rat model of PreE. Etanercept also rescues vascular pathology in the deoxycorticosterone acetate (DOCA) high-salt diet neurogenic rodent HTN model (DOCA-salt) (Guzik et al., 2007; Basting and Lazartigues, 2017), which exhibits a similar immune state to PreE (i.e. excessive pro-inflammation and inadequate anti-inflammation) (Guzik et al., 2007). Similar effects have been reported in animal models of PAH and systemic HTN (Jafri and Ormiston, 2017).

These animal models of PreE have some limitations. For instance, the RUPP rat model features placental ischemia, which occurs only in the end stage of PreE. However, the RUPP model also mirrors increased Th1, Th17, and other PreE-associated molecular changes such as increased ET-1, AT1-AA, ROS, and TNF-α, as well as critical disease phenotypes (i.e., HTN, endothelial dysfunction) (Ibrahim et al., 2017). However, given that it is an end-stage model, RUPP is limited in its capacity to represent early PreE pathogenesis, where immune based therapies may be most useful. Despite this limitation, the results in RA, AS, IBD, and PreE animal models suggest the effectiveness of anti-TNF-α treatments for treating pro-inflammation in PreE, warranting further exploration.

## TNF-α and Endothelin-1/Endothelin-1 receptor A

TNF-α is thought to exacerbate PreE by activating vasoconstrictive endothelial cells and increasing coagulation, vascular permeability, microvascular leakage, and trophoblastic apoptosis (Kaplanski et al., 1997; Huppertz and Kingdom, 2004). Resultant cellular debris activate endothelial cells via ET-1/ET-1 receptor A (ET_A_) pathway activation, causing dysfunction and eliciting further pro-inflammatory activation, vasoconstriction, and HTN (Raghupathy, 2013; Morris et al., 2016). TNF-α also stimulates ET-1 production by endothelial cells, which is elevated in the RUPP rat model (Alexander et al., 2001). Three ET_A_ antagonists (ambrisentan, bosentan, and macitentan) that inhibit vasoconstriction to prevent HTN and slow disease progression are currently approved for the treatment of PAH because of their effects on IL-17 (National Institute of Diabetes and Digestive and Kidney Diseases, 2012).

IL-17 processes also interact with ET signaling. Despite modulating IL-17 production by Th17 cells, ET/ET_A_ does not appear to directly mediate Th17 differentiation (Tanaka et al., 2014). Instead, effector T cell differentiation is promoted by DC maturation, which is in turn associated with increased ET-1 (Tanaka et al., 2014). Incubation with ET_A_ receptor antagonists inhibit DC maturation, thus downregulating mature DC stimulation of cells including Th1 and cytotoxic NK cells (Guruli et al., 2004; Tanaka et al., 2014). In both *in vitro* and *in vivo* mouse models, ET_A_ receptor blockade reduces IL-17 production following Th17 activation, which is discussed later in this review. These reductions in IL-17 release and Th17 activation are associated with significantly decreased risk for HTN, HELLP syndrome, and renal dysfunction following ischemic injury (Alexander et al., 2001; Morris et al., 2016; Boesen, 2018). By neutralizing TNF-α, there may be fewer cellular debris in circulation and in the placenta, thus blunting ET-1/ET_A_ pathway activation and limiting pro-inflammation, HTN, and renal dysfunction. Together, this may lead to less TNF-α-mediated vasoconstriction, vascular permeability, and apoptosis (Figure 2).

## CD28 Antibodies

Another cytokine-specific antibody manipulation of interest for the treatment of PreE is the superagonistic monoclonal antibody (SA) against CD28. Recently, testing of anti-CD28 monoclonal antibodies (mAbs) has been studied in PAH as a Treg stimulator because CD28 is a potent Treg activator and decreases pro-inflammatory cytokine secretion (Tabares et al., 2014). In the PreE RUPP rat model, SA mAb administration stimulated Treg cell proliferation, increasing the percent of Treg cells relative to those seen in normal pregnancy and reducing the clinical signs of PreE in response to placental ischemia (Ibrahim et al., 2017). Additionally, IL-6 and IL-2 levels, which promote Th1 proliferation and activate NK cells, were decreased to control levels in the RUPP model after SA mAb treatment (Ibrahim et al., 2017). Stimulation of cytolytic NK cells increased apoptosis and debris, aggravating the pro-inflammatory response further. NP controls maintained balanced cytokine and Treg levels, normal BP, and delivered pups of a healthy weight (Ibrahim et al., 2017). A reduction without complete inhibition is notable because maintaining appropriate pro-inflammation is essential for proper placental invasion (Sargent et al., 2006). SA mAb administration also normalizes fetal weight in RUPP offspring, highlighting the PreE-relevant benefits of Treg and NK cell stimulation. These findings offer insights into antibody-based tactics that manipulate CD4+ T (Figure 2) and NK cell mechanisms (Figure 3).

## IL-10 Administration and Induction

IL-10, which is produced by macrophages, Tregs, and regulatory NK cells, is an immunosuppressive cytokine necessary for appropriate differentiation of decidual macrophages and subsequent immune activation in trophoblast invasion and tissue remodeling (Heyward et al., 2017). Decreased IL-10 is associated with PreE and other inflammatory diseases, as IL-10 impedes macrophage differentiation and effectively suppresses pro-inflammation triggered by fetal/paternal antigens (Figures 2–4) (Lutsenko et al., 2014; Harmon et al., 2016; Heyward et al., 2017).

Animal models of PreE offer some insights into a potential role for IL-10 in PreE pathogenesis. For instance, in the BPH/5 inbred murine model of early PreE, IL-10 is spontaneously decreased. This model also develops early impaired invasion and placentation phenotypes and exhibits deficient spiral artery remodeling (Hoffmann et al., 2008). Decreased IL-10 in the BPH/5 model results from suppression of decidual macrophages and inadequate immune activation in response to fetal antigens (Davisson et al., 2002).

While the BPH/5 model represents early PreE phenotypes, the RUPP model, which recapitulates later pathoetiologic changes in PreE, demonstrates a potential therapeutic role for IL-10 supplementation in PreE. Administering IL-10 in the RUPP model leads to a partial rescue of HTN (Harmon et al., 2015). Moreover, IL-10 delivery via osmotic mini pumps in the RUPP model significantly lowers circulating Th1, Th17, and placental ROS, increases Treg cell differentiation, and normalizes TNF-α levels (Figure 2) (Harmon et al., 2015). This demonstrates that IL-10 agonism may help to prevent PreE pathogenesis via immune mechanisms. Given that IL-10-producing macrophages are decreased in PreE, together with data from the BPH/5 and RUPP models, suggests that IL-10 supplementation may be one PreE therapeutic avenue.

In addition to its utility in PreE, IL-10 supplementation and induction are possible prophylaxis and treatment options for a variety of additional pro-inflammatory diseases. For example, in adjuvant arthritis mice models for RA, IL-10 inducers or IL-10 producing cells (e.g., Th2, Tregs) attenuated clinical phenotypes and normalized IL-10 levels after 28 days of administration (Lutsenko et al., 2014). In another pro-inflammatory disorder, osteoarthritis, increased IL-10 expression significantly decreased pain in a dog model, supporting the benefits of IL-10 prophylactics for inflammatory conditions (Uzieliene et al., 2021). IL-10 also prevents PAH via suppressed antigen presentation by macrophages and DCs, blunted pro-inflammatory cytokine release, and activated Treg cells (Lai et al. (2011); Jafri and Ormiston, 2017). Incubating naïve CD4+ T cells with IL-10-producing DCs also promotes Treg cell differentiation (Harmon et al., 2015), which is decreased in PreE. Collectively, these results demonstrate that IL-10 may be a novel, effective modulator of pro-inflammatory phenotypes and disease pathogenesis across multiple conditions.

## Transfer of Tregs to Promote Anti-inflammation

Transfer of Treg cells is a direct means by which to increase Treg levels. T-cell-based immunomodulation strategies are advancing quickly in the treatment of blood and tumor malignancies, as well as other immune-mediated diseases. The use of donor and engineered Tregs to combat immune dysregulation is also being studied in IBD (Graham and Xavier, 2020; Xiao et al., 2021), DM (Bluestone et al., 2015), PAH, SLE (Dall'Era et al., 2019; Shan et al., 2020), and in rodent models of PreE (Jafri and Ormiston, 2017). Collectively, these studies demonstrate that Treg adoptive transfer is effective in promoting anti-inflammation and restoring a healthy ratio of Treg/Th2:Th1/Th17, and is not associated with significant adverse effects (Ou et al., 2018). Preclinical trials have likewise demonstrated that adoptive transfer of CD4+CD25 + Treg cells into rats with PAH reduces HTN to immunocompetent control rat levels (Jafri and Ormiston, 2017). The first adoptive Treg cell therapy was used in a patient with SLE in 2019, yielding increased Treg cell activation and decreased Th1-mediated pro-inflammation (Dall'Era et al., 2019). Moreover, a phase I trial in type 1 diabetes demonstrated that a single Treg infusion was safe, well tolerated, and led to enhanced suppressive activity due to Treg and IL-2 expansion and 1 year of Teff cytokine depletion (Bluestone et al., 2015).

As in the clinical literature, studies of Treg cellular infusion in rodent models of hypertensive disease with Treg cell infusion also demonstrate mitigated inflammatory pathology. Mouse models of HTN demonstrate a protective effect when Treg cells are adopted from healthy pregnancies to hypertensive ones. More specifically, Treg cell transfer from healthy controls to HTN mice promotes mast cell repair of placental and vascular defects and reduces BP (Maganto-García et al., 2011; Matrougui et al., 2011; Woidacki et al., 2015). However, lowering BP does not necessarily prevent PreE. For instance, in the RUPP rat model of PreE, while transfer of Tregs from a healthy pregnant rat into RUPP rats prior to uterine artery occlusion, which is used to model PreE placental ischemia, rescued multiple PreE endophenotypes (BP, growth restriction, vasoactive factors, pro-inflammation, placental oxidative stress) (Cornelius et al., 2015; Harmon et al., 2016; Jafri and Ormiston, 2017; Cornelius, 2018; Robertson et al., 2019). Yet, it did not fetal morbidity (Cornelius et al., 2015). The prophylactic potential of Treg transfer in PreE is mostly reliant on a direct increase in anti-inflammatory CD4+ T cell levels (i.e., Th2 and Treg) upon treatment (Figure 2).

Adoptive Treg cell transfer remains a promising area of study because it may mitigate gestational immune dysregulation at the outset, before PreE pathology progresses. While preclinical work has demonstrated the promise of adoptive Treg cell transfer for the treatment of PreE, more research is needed to better understand the side effects, underlying mechanisms, and application of this approach to human disease.

## Decreasing IL-6 Levels

IL-6 contributes to pro-inflammation in PreE by inducing Th17 and cytotoxic T cell differentiation and inhibiting immunosuppressive Tregs and Th2 (Redman et al., 1999). Decreasing IL-6 via anti-IL-6 mAbs or toll-like receptor (TLR) five inhibitors may reduce pro-inflammation (Narazaki et al., 2017) (Figure 2), thereby modulating PreE pathogenesis. IL-6 inhibitors [e.g., anti-IL-6 receptor mAbs (e.g., sarilumab, tocilizumab) and anti-IL-6 mAbs (i.e., siltuximab)] may direct the expansion of inexperienced CD4+ T cells away from inflammatory Th1 and Th17 subtypes (Narazaki et al., 2017; Xiao et al., 2021). Clinical studies support the successful use of immunomodulation to modulate IL-6, as in IBD (Trivedi and Adams, 2018), RA (Narazaki et al., 2017), and AS (Tousoulis et al., 2016; Ridker et al., 2017). Various mAbs and TLR five antibodies have been used to achieve IL-6 regulation. The administration of these antibodies reduces IL-6 and lowers cardiovascular risk (Ridker et al., 2017). Applying similar IL-6-reduction strategies to the treatment of PreE may drive differentiation and proliferation of naïve CD4+ T cells towards an anti-inflammatory Treg and Th2 cell fate rather than a pro-inflammatory Th1 and Th17 one.

## Blocking IL-17 and Decreasing Th17

Another potential inflammatory target for the treatment of PreE is Th17, levels of which are increased in the disease. Suppression of Th17 via IL-17 recombinant receptor administration decreases HTN, oxidative stress, and AT1-AA production (Cornelius et al., 2013). A recent study utilizing chronic administration of recombinant mouse IL-17 receptor C (IL-17RC), a soluble receptor that blocks IL-17, to RUPP rats reported normalized total placental and cytolytic NK cells, decreased circulating IL-17, decreased circulating and placental Th17s, and decreased TNF-α, as well as decreased mean arterial pressure (MAP) (Travis et al., 2020). Additionally, repeated acute administrations of IL-17RC diminished Th17 proliferation (Travis et al., 2020). These studies reveal the potential efficacy of IL-17RC for treating IL-17-mediated pro-inflammation in PreE.

In the DOCA-salt neurogenic rodent model of HTN, Th17, and IL-17 levels are elevated as they are in PreE (Amador et al., 2014; Basting and Lazartigues, 2017). When given to DOCA-salt rats, Spironolactone competitively binds aldosterone and prevents Th17 activation, normalizing IL-17 levels and inducing Treg differentiation by increasing forkhead box P3 transcription (Amador et al., 2014). Administration of anti-IL-17 antibodies in the DOCA-salt model ameliorated HTN, oxidative stress, and fibrosis, likely by decreasing Th17 polarization (Amador et al., 2014). Similarly, in the HELLP rodent model of PreE, decreasing IL-17 by blocking the ET_A_ receptor preferentially reduced Th17 differentiation and lowered BP (Morris et al., 2016). Furthermore, Th17s may promote NK activation via IL-17 induction (Travis et al., 2020), though the exact mechanism underlying this remains unclear. By targeting Th17 or blocking IL-17 in PreE, pro-inflammatory cytokine production and NK cell activation may be decreased, resulting in less cytotoxic activity and thereby decreased pro-inflammation (Figure 2).

## Pravastatin

Recently, statins have attracted interest as targets for PreE prevention due to their potent anti-inflammatory effects. Statins are 3-hydroxy-3-methylglutaryl coenzyme A (HMG-CoA) reductase inhibitors that are approved by the FDA for cholesterol reduction, though they are currently contraindicated for use in pregnancy. The clinical PRINCE [pravastatin inflammation/reduced C-reactive protein (CRP) evaluation] trial confirmed that pravastatin has anti-inflammatory effects in primary and secondary prevention settings in addition to its lipid-lowering effects (Albert et al., 2001). Given these results, statins began to be considered for the treatment of pro-inflammatory diseases such as DM (van de Ree et al., 2003), metabolic syndrome (Bulcão et al., 2007), atherosclerosis (S. Antonopoulos et al., 2012), and multiple sclerosis (Youssef et al., 2002). Multiple trials have demonstrated reduced CRP and circulating pro-inflammatory cytokines with statins. *In vivo* studies have further shown specifically that administration of statins induces Th2 cytokines (including IL-4, IL-5, IL-10, and TGF-β) and suppresses Th1 cytokines (including IL-2, IL-12, TNF-α, and IFN-γ) (Youssef et al., 2002; Esteve-Valverde et al., 2018). Applying this to PreE may help balance the elevated Th1:Th2 ratio characteristic of the disease.

Despite being contraindicated for use in pregnancy, a recent study in DBA/2-mated CBA/J mice, a well-studied spontaneous abortion and fetal growth restriction mouse model (Redecha et al., 2009), demonstrated that pravastatin effectively rescued placental dysfunction and prevented fetal resorptions (Redecha et al., 2009). Another small study in a novel lentiviral vector-mediated placental sFlt-1 expression mouse model of PreE reported decreased blood pressure and proteinuria with daily pravastatin (Kumasawa et al., 2011). Several studies in pregnant mice demonstrated reduced sFlt-1 levels and reduced hypoxia, significantly increased pro-survival/antiapoptotic factors, and upregulated PLGF and VEGF-A after treatment with adenovirus-carrying sFlt-1 and statins (McDonnold et al., 2014; Saad et al., 2014; Saad et al., 2016).

Reduced sFlt-1 expression is another potential intervention strategy for PreE, which can increase PLGF and VEGF. Additionally, the use of T-NPsisFLT1 or PEG-PLA nanoparticles as sFLT1 siRNA placenta specific delivery systems can decrease sFlt-1 in pregnant CD1 mice and silencing sFlt-1 in this way is being studied as a method to ameliorate PreE in similar manners to statins (Li et al., 2020). Furthermore, a 2016 pilot randomized controlled trial assessing the use of pravastatin in PreE found no safety risks (Costantine et al., 2016), while another recent study reported lower rates of PreE and preterm delivery in high-risk individuals treated with pravastatin (Costantine et al., 2021). These results, in conjunction with the known immunomodulatory effects of statins (Figure 2), justify further immune research into pravastatin’s use in PreE prevention and therapy.

## 17-Hydroxyprogesterone Caproate

Another potential avenue for immunologic treatment of PreE is targeting cytolytic placental NK cells early in gestation to decrease Th1/17 and increase Treg/Th2 cells. Within circulation and in the placenta in PreE, non-cytolytic, type 2 NK cells are decreased while cytolytic, type 1 NK cells are increased (Fukui et al., 2012), increasing apoptosis and therefore the production of debris. Elfarra *et al.* tested 17-hydroxyprogesterone caproate (17-OHPC) in the RUPP rat model of PreE (Elfarra et al., 2020) and found that the synthetic hormone, which is commonly used to prevent preterm delivery in healthy pregnancies, has anti-inflammatory and vasodilatory effects (Dodd et al., 2013). Because the naturally occurring hormone progesterone reduces cytotoxic NK cell activity in the uterus (Dosiou and Giudice, 2005; Elfarra et al., 2020), 17-OHPC may act similarly (Elfarra et al., 2020).

In the RUPP model, 17-OHPC normalized HTN and pup weight, decreased fetal demise rates, reduced uterine artery resistance, and increased circulating and placental Th2 cells while suppressing pro-inflammatory CD4+ T cells and cytokines to restore immune balance (Elfarra et al., 2020). Circulating Th2 cells were significantly increased in RUPP rats with 17-OHPC treatment (Elfarra et al., 2020). Furthermore, cytolytic NK cells were also decreased significantly with 17-OHPC administration, and the administration was associated with attenuated hypertension in response to placental ischemia (Elfarra et al., 2020). While there is limited data on 17-OHPC prevention of PreE, phenotypes were decreased in the RUPP model (e.g., decreased total and cytolytic placental NK cells, attenuation of pro-inflammatory T cells and cytokines, decreased HTN and fetal weight), which is likely attributable to suppressed inflammation and increased placental Th2. Together, these data on Th2-NK cell dynamics suggest that hormone strategies are another potential CD4+ T cell-based PreE prevention mechanism (Figure 2).

## Treg Cell Stimulation via NK Cells and DCs

Suppressing Teff cells and manipulating NK and DC pathways are additional methods for the treatment of AS, PAH, and SLE (Xiao et al., 2021). PreE pregnancies have increased populations of cytolytic, type 1 NK cells, which also exhibit enhanced cytolytic activation (Fukui et al., 2012; Sargent et al., 2007). These cytolytic NK cells secrete increased levels of TNF-α (Fukui et al., 2012), which contribute to PreE pathogenesis. Conversely, however, non-cytolytic, type 2 NK cells also stimulate Treg proliferation by releasing interferon gamma (IFN-γ) (Fukui et al., 2012). Therefore, normalizing NK levels may provide another route for PreE prevention. While the dynamics and direct interactions between NK cells, DCs, and resultant Treg stimulation require further interrogation, some promising manipulations inhibit cytotoxic NK cells and others prevent DCs from presenting self-antigens (Bayry et al., 2003; Gu et al., 2012; Elfarra et al., 2020).

DCs and other antigen presenting cells (APCs) also contribute to immune dysregulation by presenting fetal antigens and stimulating pro-inflammatory Th1 and Th17 cells at the maternal-fetal interface. It is likely that modulation of abundant decidual NK cells and DCs via interactions with T cells and their cytokine/chemokine products, may be one avenue for PreE treatment and prevention. In fact, decidual NK cells are a potential target for early pregnancy pathology and immune dysregulation, for example in recurrent miscarriage or implantation deficits (Tao et al., 2021). However, the precise interactions between CD4+ T cells and decidual NK cells at the maternal-fetal interface requires further elucidation.

For example, IL-10 and TGF-β (produced by Tregs), as well as Treg surface molecules such as inhibitory receptor cytotoxic T lymphocyte-associated antigen-4 (CTLA-4) inhibit DC function and maturation (Figure 4). This is likely because CTLA-4 binds to CD80/CD86 and blocks the activation of naïve T cells by DCs (Gu et al., 2012). DCs mediate either immune rejection or tolerance because they direct T cell alloresponses towards pro- or anti-inflammatory subtypes (Mashreghi et al., 2008). Stimulating the production or adoptive transfer of immature DCs can be manipulated for prevention use to prevent downstream activation of pro-inflammatory T cells (Figure 1) or cytolytic NK cells (Figure 4). CTLA-4, IL-10, and TGF-β and their subsequent inhibition of DC function are associated with increased Treg numbers and decreased pro-inflammatory responses in AS (Dietrich et al., 2012; Foks et al., 2015; Ou et al., 2018). The immunological similarities between AS and PreE suggest that DC inhibition decreases pro-inflammatory Th1 and Th17 differentiation, preventing PreE pathogenesis.

Another DC modulator is cobalt protoporphyrin induced heme oxygenase 1 (HO-1), which is currently used to protect against inflammatory liver failure and ischemia reperfusion injury after transplant procedures. This is because HO-1 has potent anti-inflammatory properties; it attenuates the expression of various proinflammatory genes, such as TNF-α, IL-1, and -6 by mononuclear phagocytes and downregulates leukocytes adhesion in response to oxidative stress (Mashreghi et al., 2008; Vijayan et al., 2010). Similarly, intravenous immunoglobulin G administration is used as a therapy for many immune-mediated conditions, transplantation, and systemic inflammatory diseases (e.g., SLE, Guillain-Barre syndrome, RA, and more) because it inhibits DC maturation, increases anti-inflammatory IL-10, and down-regulates pro-inflammatory T cells (Bayry et al., 2003) (Figure 4). These approaches, which decrease NK cell numbers and blunt DC maturation, may demonstrate similar effectiveness in restoring a proper immune balance in PreE and therefore deserve further exploration.

## Discussion

A healthy pregnancy requires a balance of pro- and anti-inflammatory immune factors to accomplish proper placentation and protect the pregnant person from invading pathogens, while also establishing tolerance to fetal antigens. Growing evidence indicates that PreE pathology is in part attributed to an altered immune response; namely elevated pro-inflammatory Th1/Th17 and reduced anti-inflammatory Treg/Th2, along with their respective cytokines (Redman et al., 1999; Redman and Sargent, 2010). A dysregulated inflammatory response is associated with poor invasion and inadequate remodeling of the uterine spiral arteries during stage one of the classic Two Stage model of PreE (Redman, 1991; Redman et al., 1999). There is subsequent placental oxidative stress and hypoxia, and Stage 2 follows, prompting further pro-inflammation and ultimately the clinical manifestations of PreE (HTN, proteinuria, and end-organ damage) (Redman et al., 1999; Committee on Practice Bulletins-Obstetrics, 2020). Though the exact mechanism driving this immune shift is not fully understood, there is compelling evidence that maladaptation to fetal/paternal alloantigens is to blame. Immune modulation is thus a prime goal of successful PreE prophylactics (Li and Wi, 2000; Kho et al., 2009; Redman and Sargent, 2010).

Immunomodulation in other immune-mediated diseases to improve the immunosuppressive capacity of Tregs and decrease Th1/Th17 activation may offer viable strategies for the treatment and prevention of PreE. Specifically, immunomodulators targeting CD4+ T cell mechanisms are emerging as potential drugs of interest for PreE. Together, the studies and findings reviewed here point to a promising role for modulation of Tregs, Th1, and Th17 cells, in particular by specific cytokine inducers or suppressors. Existing classes of drugs (e.g., statins, spironolactone) may also be harnessed to achieve cytokine/chemokine modulation and thereby CD4+ T cell attenuation to treat and prevent PreE. The weight of the evidence also demonstrates that specific modulators (e.g., anti-IL-17) may be less effective than more global immunomodulatory agents (e.g., anti-CD28) in reducing the burden of pro-inflammatory CD4+ T cells in PreE pathogenesis.

Application of approved therapies for other immune-mediated diseases to the treatment of PreE has likewise yielded promising results. While most studies aim to normalize immune dysregulation by stimulating Treg cells and inhibiting Th1 and Th17 cells via cytokine induction or suppression of their downstream activation by mature DC or apoptotic debris (due to NK cells), adoptive transfer of Treg cells, or neutralization of pro-inflammatory cytokines via antibody-based drugs also offers promise. Furthermore, Treg therapies have gained popularity for use in combatting inflammatory and immune diseases. Over 50 total registered clinical trials using Tregs occurred in 2019: 12 for autoimmune diseases, 23 for hematopoietic stem cell transplantation or graft-versus-host disease, and 16 for solid organ transplantation (Ferreira et al., 2019). PreE pregnancies have striking commonalities to these disease categories. Because the immunological mechanisms underlying PreE are likely present very early in pregnancy, targeting CD4+ T cell pathways provides a possible and very promising avenue for PreE prevention, as this review demonstrates. Ultimately, by normalizing the maternal response to fetal antigens and the broader immune state (e.g. restoring the proper Th1/17:Treg/Th2 cell ratio), PreE pathogenesis may be interrupted.

Despite some successes, the application of specific immunomodulatory tactics to PreE is currently limited due to poor chemical stability, high cost, regulatory restrictions, and poor patient compliance with repeated dosing protocols (Xiao et al., 2021). However, existing work in other pro-inflammatory disorders offer important insights into the treatment of PreE, a devastating disease that impacts nearly 6.6 million pregnancies globally each year (Duley, 2009). It is essential that research continue to develop and assess improved or novel approaches to treating and preventing the immunology of PreE.
